# Is Regenerative Medicine Ready for Prime Time in Diabetic Polyneuropathy?

**DOI:** 10.1007/s11892-018-0971-y

**Published:** 2018-01-30

**Authors:** Tatsuhito Himeno, Hideki Kamiya, Jiro Nakamura

**Affiliations:** Division of Diabetes, Department of Internal Medicine, Aichi Medical University, 1-1 Yazakokarimata, Nagakute, Aichi 480-1195 Japan

**Keywords:** diabetic polyneuropathy, mesenchymal stromal cell, endothelial progenitor cell, induced pluripotent stem cell, cell transplantation, regenerative medicine

## Abstract

**Purpose of Review:**

After a prolonged warm-up period of basic research, several modalities of cell replacement therapies are under development for diseases with no available cure. Diabetic polyneuropathy (DPN) is one of the most prevalent chronic diabetes complications that causes sensorimotor dysfunction, subsequent high risks for lower limb amputations, and high mortality. Currently, no disease modifying therapy exists for DPN.

**Recent Findings:**

Several types of well-documented stem/progenitor cells have been utilized for cell transplantation therapies in DPN model rodents: mesenchymal stromal cells (MSCs), endothelial progenitor cells (EPCs), and cells with similar characteristics of MSCs or EPCs derived from embryonic stem cells or induced pluripotent stem cells. Some recent experimental studies reported that these immature cells may have beneficial effects on DPN.

**Summary:**

Although the role of nerve regeneration in the pathology of DPN has not been sufficiently elucidated, many intervention studies attempting regenerative therapy of DPN have been reported. Further studies are needed to better evaluate the potential of regeneration in reversing the pathology of DPN

## Introduction

Today, the field of regenerative medicine is undergoing great development.Traditionally, there have been several options for tissue regeneration, i.e., artificial scaffold, cell transplantation, and replacement of various cytoprotective or growth factors. However, as new cell sources including induced pluripotent stem cells (iPSCs) [1, 2••] were introduced and many clinical trials using mesenchymal stromal cells (MSCs) [3–5] or other progenitor/stem cells were successively accumulated [6, 7], the potential for cell transplantation therapies seems to be expanding very rapidly. In this review, we discuss current evidence on the potential of cell transplantation therapies as regenerative medicine for diabetic polyneuropathy (DPN).

**Fig. 1:**
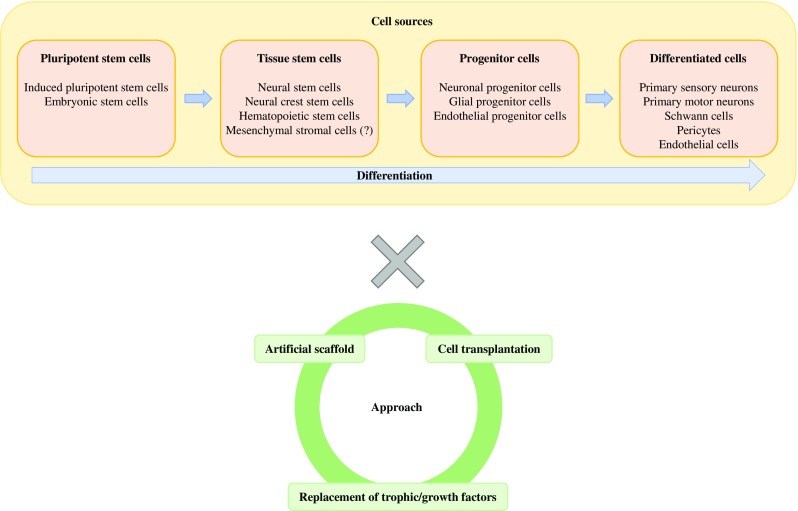
Strategy of cytotherapy in diabetic polyneuropathy

An overview outlining proposed steps to develop this new therapeutic option for DPN is presented in Fig. 1.

## Regenerative Medicine with MSCs

Adult stem cells or tissue stem cells are cells distributed in various tissues and organs throughout our adult lives. It is generally accepted that hematopoietic stem cells, neural stem cells, and intestinal stem cells can be regarded as stem cells [8–12] because they possess self-renewal abilities and multiple differentiation potentials [13–16]. Meanwhile, as bone marrow derived MSCs were also long considered to be stem cells [17] and are not susceptible to malignant transformation [18, 19], researchers have attempted to utilize them in the treatment of various diseases [20]. As a consequence, focusing on immunomodulatory effects of MSCs, intravenous infusion of MSCs has already been approved for clinical uses in graft-versus-host diseases following hematopoietic stem cell transplantation. In addition to bone marrow, MSCs are also derived from various other tissues, such as dental pulp [21], adipose tissues [22], umbilical cords [23] and placentae [24], and have been reported to have similar properties of multipotent differentiation.

Common features of these MSCs are adherence to plastic dishes, and the ability to differentiate into mesodermal cells in, for example, bone, cartilage, and adipose tissues [17, 25]. However, as MSCs comprise heterogeneous subsets expressing various biomarkers, it is still difficult to verify a specificity of MSCs [26, 27]. Morphology and combinations of cell surface markers may identify the MSCs [28, 29].

However, it is not yet established that MSCs are true stem cells because their proliferation is mostly self-limited and their self-replication ability is scarcely verified [18, 30, 31]. Moreover, as the in vivo distribution and physiological roles of MSCs are not fully elucidated, the stemness and roles of MSCs are still controversial [32•, 33]. The embryological origin of MSCs is also unclear. As MSCs can differentiate to neurons and glias and express some neural markers, an neuroectodermal origin is possible, although not fully established [34].

As described above, MSCs are expected to behave as progenitor-like cells, i.e., to promote tissue reconstruction by providing an extracellular matrix, to exert cytoprotective actions through production of various growth factors, and to accelerate cell proliferation. These features imply possible usefulness in the whole body; therefore, clinical trials have been considered for a wide variety of diseases.

As of June 2017, on the WHO’s International Clinical Trials Registry Platform, 651 trials were retrieved in response to the search query "mesenchymal stem cell(s)", and 89 trials with "mesenchymal stromal cell(s)".Although the tissue used for the isolation of MSCs has conventionally been bone marrow, most current studies utilize placentae, umbilical cords, or adipose tissues to obtain MSCs. This expansion of resource options has allowed for a rapid increase in clinical trials around the world. At the moment, as briefly mentioned above, MSCs are used in clinical settings against GVHD associated with bone marrow transplantation in Canada, New Zealand, and Japan. On the other hand, no application as regenerative medicine has been achieved except for a phase III trial for Crohn's disease, which is being carried out in the United States of America.

## Cytotherapy Using MSCs in DPN

Some basic research studies have shown that bone marrow-derived MSCs have improved nerve conduction velocities in streptozotocin-induced diabetic rats [35, 36]. These reports have suggested the possibility that paracrine effects of growth factors such as vascular endothelial growth factor (VEGF) and fibroblast growth factor-2 (FGF-2), produced by MSC-ameliorated DPN, may be derived from exosomes [37, 38], but the beneficial effects of exosomes on DPN has not been yet verified.

The differentiation potential of MSCs into Schwann cells may be an additional argument for their potential benefit in the treatment of DPNb [39, 40], in that a direct supply of neural cells derived from MSCs may contribute to the regeneration of the peripheral nervous system (PNS).

In addition, MSCs also have the ability to differentiate into pericytes of capillaries, which suggests that MSCs might aid angiogenesis in numerous tissues and organs. However, as the potential for differentiation can decrease with aging of donor cells [41], the experimental conditions need to be thoroughly studied in order to obtain stable results.

Although cell transplantation therapies are in full bloom in this decade, it should be noted that cytotrophic/growth factors also attracted attention in experimental medicine. Some growth factor replacement therapies in DPN using nerve growth factor [42] or VEGF [43] proceeded to clinical trials, but although these replacements achieved a certain level of beneficial effects on DPN, they unfortunately were not viewed as having a wide application in clinical settings. It has also been shown that intramuscular administration of FGF-2 increased sciatic nerve blood flow and improved nerve conduction velocities in diabetic rats [44]. As mentioned above, it has been reported that MSCs also express various growth factors including neurotrophic and angiogenic factors, e.g., NGF, FGF-2, and VEGF. Therefore, MSCs are expected to function as a composite provider of these cytoprotective factors. Currently, only one clinical study using MSCs for DPN has been registered in the ClinicalTrials.gov database (NCT02387749), but although the trial has already concluded, the results have not yet been published.

Although a bright future is anticipated for the use of MSCs, some problems need to be addressed. First, the existence of MSCs is still questionable. In 2008, Crisan et al. proved that non-cultured human pericytes expressed MSC markers and exhibited the potentials of osteogenic, chondrogenic and adipogenic differentiation [45]. On the other hand, Guimarães-Camboa et al., recently reported that pericytes did not behave as MSCs in vivo [32•]. They suggested that MSCs arose from ex vivo manipulations of perivascular cells. Second, the definitions of MSCs both in humans and rodents still depend on artificial conditions: adherence to the surface of hard dishes; multiple differential inducibility into osteocytes, chondrocytes and adipocytes; and expression of cell surface markers including CD146 and PDGF-Rβ. Despite a broad and lengthy employment of this combinative definition of MSCs, MSC proliferation ability differs in each paper from several weeks to an infinite time [46, 47] and a variety of cell marker sets is also utilized. Third, the pathophysiological contribution of MSCs to various diseases has never been clarified in any experimental or clinical studies. Given that the physiological roles of MSCs in normal tissues have not been fully described, due to the lack of identity of MSCs in vivo, the indispensability of MSCs in pathologies of related diseases has not also been described. Limited information is available to discuss the role of MSCs in pathological changes of DPN, but we previously proved the reduction of CD29 positive/CD90 positive bone marrow mononuclear cells, in which MSCs are contained, in diabetic rats compared with non-diabetic rats [48]. However, this population reduction is just indicative of, but not convincing evidence of, the impact of MSCs in the pathology of DPN. Given these limitations, further investigations to elucidate the physiology of and to define MSCs are recommended in the future.

## Transplantation of Endothelial Progenitor Cells in DPN

Like MSCs, vascular endothelial progenitor cells (EPCs) have a wide target range of diseases in the field of regenerative medicine. EPCs were first reported as CD34 positive cells in the mononuclear cells derived from the bone marrow by Asahara and Murohara et al., in 1997 [49]. Since the definition and existence of EPCs has been called into question [50], the distribution and physiological roles of stem or progenitor cells responsible for angiogenesis in adults is currently being actively discussed [51]. No clinical application using EPCs has been actualized despite numerous clinical trials having been registered. Therefore, in order to consider effective clinical applications, it will be necessary to investigate the biology of EPCs in more depth and to revisit the definition of EPCs.

Regardless of the lack of validated EPCs, many basic studies utilizing EPCs have been conducted. Naruse et al., reported that the transplantation of umbilical cord blood-derived EPCs improved the capillary-muscle fiber ratio in soleus muscle and nerve conduction velocities in a rat DPN model [52]. Another group also indicated that some of the beneficial effects on DPN were delivered from angiogenic and neuroprotective factors produced by EPCs [53]. According to the report by Jeong [53], engrafted EPCs were preferentially localized along the course of the vasa nervorum of sciatic nerves in diabetic mice. However, the paper referred to bone marrow-derived adherent cells as EPCs, thus the transplanted EPCs would consist of heterogeneous populations. Therefore, it is hard to conclude that the beneficial effects were exclusively demonstrated by EPCs themselves.

## Pluripotent Cells as a Source for Regenerative Therapy of DPN

Owing to the engineering breakthrough of pluripotent cells including embryonic stem cells (ESCs) and induced pluripotent stem cells (iPSCs), several types of immature cells that can differentiate into mature cells comprising the PNS were derived from pluripotent cells, and employed for DPN treatment in experimental studies. We previously reported that the transplantation of angioblast-like cells derived from murine ESCs increased capillary density in the soleus muscle and improved motor and sensory nerve conduction velocities (NCVs) in diabetic mice [54]. As an alternative approach to regeneration of the PNS, we utilized neural crest stem cell-like (NCSC-like) cells derived from murine iPSCs. The NCSC-like cells were engrafted into muscles of the lower limbs; after their intramuscular transplantation, they differentiated into pericytes and S100-positive Schwann-like cells. The transplantation of NCSC-like cells also ameliorated perception functions and NCVs of DPN model mice [55]. Furthermore, MSC-like cells derived from iPSCs were also employed for the regenerative therapy in DPN [56]. The MSC-like cells provided not only pericytes but also Schwann cells, and some engrafted Schwann-like cells surrounding neuronal axons in sural nerves.

## Regenerative Capacity in DPN

Many tissue engineering materials have been proposed for wound healing in diabetic animals, particularly on diabetic foot ulcers [57]. Furthermore, regenerative medicine has also been recognized to promote neural axon recruitment using artificial materials in peripheral nerve injury models in diabetic animals [58]. However, it has not been attempted to perform the regeneration of axons using a particular scaffold in the DPN model. In addition, it is indisputable that the replacement of cell bodies of sensory neurons themselves in dorsal root ganglions (DRGs) utilizing scaffolds mimicking a structure of DRGs has never been attempted. The reason why these experiments have never been attempted is because the regenerative capability in the PNS may be limited only to axons, especially those on the periphery of nerves. Since peripheral nerves are anatomically separated from systemic blood circulation by the perineurium and the blood-nerve barrier, it is possible that therapeutic interventions act only on the nerve terminals and their vicinity.

The potential roles of Schwann cells and vascular cells in the pathophysiology of DPN have not been taken sufficiently into consideration. It is assumed that these cells would promote regeneration of axons or blood vessels in the PNS.

Therefore, although most glial or vascular progenitor/stem cells have been injected in intravenous or intramuscular fashion [35], the validity of the transplantation route has never been considered due to a scarcity of basic knowledge about the behavior of these cells and their derivatives. It is necessary to clarify in detail how regeneration occurs in the PNS of DPN in the future. On that basis, the way to realization of regenerative medicine will be developed. In other words, we should return to rigorous investigation of the mechanism of homeostasis of the PNS and regeneration in DPN, and thereafter, the pathophysiology of DPN would be further clarified.

## Conclusion

Although regenerative therapies of DPN are hampered by an insufficient insight into the pathology of DPN, numerous trials that might be slightly speed-before-quality have been presented. After revealing the regeneration mechanism of the PNS in DPN, several types of cells including vascular, nervous, and/or mesenchymal cells could be considered for an application in regenerative medicine.
